# Discovery and function exploration of microRNA-155 as a molecular biomarker for early detection of breast cancer

**DOI:** 10.1007/s12282-021-01215-2

**Published:** 2021-01-21

**Authors:** Xuemin Liu, Qingyu Chang, Haiqiang Wang, Hairong Qian, Yikun Jiang

**Affiliations:** Department of General Surgery, Zhangjiagang TCM Hospital Affiliated to Nanjing University of Chinese Medicine, Kang Le Road No. 4, Zhangjiagang, 215600 Jiangsu China

**Keywords:** Breast cancer, MicroRNA-155, Diagnosis, Biomarker

## Abstract

**Background:**

MicroRNA-155 (miR-155) may function as a diagnostic biomarker of breast cancer (BC). Nevertheless, the available evidence is controversial. Therefore, we performed this study to summarize the global predicting role of miR-155 for early detection of BC and preliminarily explore the functional roles of miR-155 in BC.

**Methods:**

We first collected published studies and applied the bivariate meta-analysis model to generate the pooled diagnostic parameters of miR-155 in diagnosing BC such as sensitivity, specificity and area under curve (AUC). Then, we applied function enrichment and protein–protein interactions (PPI) analyses to explore the potential mechanisms of miR-155.

**Results:**

A total of 21 studies were finally included. The results indicated that miR-155 allowed for the discrimination between BC patients and healthy controls with a sensitivity of 0.87 (95% CI 0.78–0.93), specificity of 0.82 (0.72–0.89), and AUC of 0.91 (0.88–0.93). In addition, the overall sensitivity, specificity and AUC for circulating miR-155 were 0.88 (0.76–0.95), 0.83 (0.72–0.90), and 0.92 (0.89–0.94), respectively. Function enrichment analysis revealed several vital ontologies terms and pathways associated with BC occurrence and development. Furthermore, in the PPI network, ten hub genes and two significant modules were identified to be involved in some important pathways associated with the pathogenesis of BC.

**Conclusions:**

We demonstrated that miR-155 has great potential to facilitate accurate BC detection and may serve as a promising diagnostic biomarker for BC. However, well-designed cohort studies and biological experiments should be implemented to confirm the diagnostic value of miR-155 before it can be applied to routine clinical procedures.

## Background

Breast cancer (BC) remains the most prevalent cause of cancer mortality in females worldwide [1]. Like all other malignant tumors, the prognosis of BC is highly associated with the extent how early this disease is diagnosed [2]. Currently, several conventional methods, including imaging examination and biopsy, and some tumor markers such as CEA and CA15-3 have been confirmed to be beneficial for detection of BC [3]. Nevertheless, there are some deficiencies as they are invasive and harmful procedure or not sensitive enough for accurate BC diagnosis. Therefore, the development of efficient diagnostic methods or tumor biomarkers for BC is urgently needed.

MicroRNAs are a class of 18–25 nucleotide, non-coding RNAs that regulate gene expression through targeting messenger RNAs and triggering either translational repression or RNA degradation [4]. Increasing studies have demonstrated that microRNAs were highly involved in various physiological and pathological processes such as cell proliferation, differentiation, and apoptosis [5]. Moreover, a large number of articles have proved that the abnormal expressions of microRNAs have a direct association with the development and progression of various cancers including BC. Subsequent evidence also indicated that circulating microRNAs were highly stabile and could be easily extracted and measured, revealing that circulating microRNAs might serve as promising biomarkers for early detection of BC [6].

Among a wide spectrum of microRNAs, microRNA-155 (miR-155) has gained great attention and numerous studies have revealed the critical role of miR-155 in the development of some cancers including colorectal cancer, oesophageal squamous cell carcinoma, lung cancer and BC [7]. Recently, several studies have revealed that aberrant expression of miR-155 may become a potential diagnostic marker of several types of cancers including BC [8]. Nevertheless, the diagnostic accuracy of miR-155 in BC was inconsistent or even contradictory among different studies. In addition, the biomarker roles of miR-155 during the initiation and progression of BC are still poorly illustrated by previous studies.

Thus, we first performed a meta-analysis, to synthesize available evidence on miR-155 as a diagnostic biomarker in patients with BC. Furthermore, we designed a bioinformatics study to uncover the potential biomarker roles of miR-155.

## Methods

### Literature search strategy

To identify the relevant studies, we searched through the online PubMed, EMBASE and Web of Science databases until May 1, 2020. The key words used for literature retrieval were as follows: (“microRNA-155” OR “miR-155” OR “miRNA-155”) and (“breast cancer” OR “breast tumor” OR “breast carcinoma” OR “breast neoplasm”). The references of all the relevant publications were manually searched to obtain additional eligible studies.

### Inclusion and exclusion criteria

Studies were considered eligible if they met the following criteria: (1) all the patients with BC must have been confirmed by pathological examination; (2) the levels of miR-155 were measured; (3) they presented sufficient data to allow the establishment of two-by-two tables, including true positives (TP), false positives (FP), true negatives (TN), and false negatives (FN).

Studies were excluded if they were: (1) reviews, meta-analysis, letters, commentaries, or abstracts presented in conferences; (2) lacking sufficient data for calculation of specificity and sensitivity with 95% confidence intervals (CIs); (3) duplication of previous publications.

### Data extraction and quality assessment

Two investigators (Xuemin Liu and Qingyu Chang) independently extracted all the data from eligible studies. A third investigator (Haiqiang Wang) was responsible for reconciling disagreements when the results were controversial. The extracted data elements of this study for diagnosis included: the first author’s name, country of research, year of publication, age of patients, ethnicity, sample size, sample source, detection method, sensitivity, specificity, TP, FP, FN, and TN. We estimated the quality of each study using the revised Quality Assessment of Diagnostic Accuracy Studies (QUADAS-2), which has been confirmed to be an effective tool for assessing the quality of diagnostic tests [9].

### Statistical analysis

The data analysis to assess the diagnostic role of miR-155 in BC was performed by STATA 14.0. The bivariate meta-analysis model was applied to obtain the pooled sensitivity, specificity, positive likelihood ratio (PLR), negative likelihood ratio (NLR), and diagnostic odds ratio (DOR) [10]. Furthermore, the summary receiver operator characteristic (SROC) curve was generated based on pooled sensitivity and specificity of included studies and the corresponding area under the SROC curve (AUC) was calculated to estimate the accuracy of miR-155 in BC detection [11]. Statistical heterogeneity was evaluated using Cochran-Q test and *I*^2^ test. *P* value < 0.05 for Cochran’s *Q* test, or I^2^ > 50%, suggested an existence of significant heterogeneity [12]. The Spearman correlation coefficient of logarithm sensitivity and 1-specificity was calculated for measuring the threshold effect. In addition, subgroup, meta-regression and sensitivity analyses were employed to investigate the potential sources of heterogeneity among included studies. Finally, publication bias was checked with the Deeks’ funnel plot [13]. A *P* value less than 0.05 for two-tailed was considered statistically significant.

### Target genes prediction of miR-155

We then performed a bioinformatics analysis to explore the potential function of miR-155. First, the microRNA prediction data were downloaded from the microRNA target gene prediction website miRTarBase, which has been one of the most comprehensive databases with experimentally validated microRNA–target interactions [14]. We only selected the human microRNA–target information for further analysis.

### GO enrichment and pathway analysis

Gene ontology (GO) analysis was carried out for annotating the parental genes according to the biological processes (BP), cellular components (CC) and molecular functions (MF) [15]. Besides, we provided pathway enrichment analyses for miR-155 targets using the Kyoto Encyclopedia of Genes and Genomes (KEGG) database [16]. To enable better recognition of the biological functions of miR-155, we used the online tool Database for Annotation, Visualization and Integrated Discovery (DAVID) to perform GO enrichment and KEGG analyses [17]. *P* value < 0.05 was considered statistically significant.

### Establishing the PPI network, selection of hub genes and module analysis

To identify the hub regulatory genes and to investigate the interactions among the target genes of miR-155, a protein–protein interaction (PPI) network analysis was conducted. The Search Tool for the Retrieval of Interacting Genes (STRING) is a biological database constructed for collecting PPI information, and then analyzing the functional interactions between proteins [18]. All the targets of miR-155 were uploaded onto STRING to retrieve their PPI data and only the interactions with a combined score > 0.4 validated by experiments or collected by text mining were set to be the cutoff criterion. Afterwards, the PPI network was visualized with Cytoscape software [19]. The CytoNCA plug-in of Cytoscape was used to identify the hub genes from PPI network, and the hub genes were selected according to three different centrality measures, containing betweenness centrality, closeness centrality and degree centrality [20]. Furthermore, the plug-in of Molecular Complex Detection (MCODE) in Cytoscape software was employed to search the significant modules in PPI network [21]. Finally, KEGG pathway enrichment analyses were performed with the hub genes and genes involved in the selected modules. The threshold *P* value < 0.05 was considered statistically significant.

## Results

### Descriptive assessment and study characteristics

**Fig. 1:**
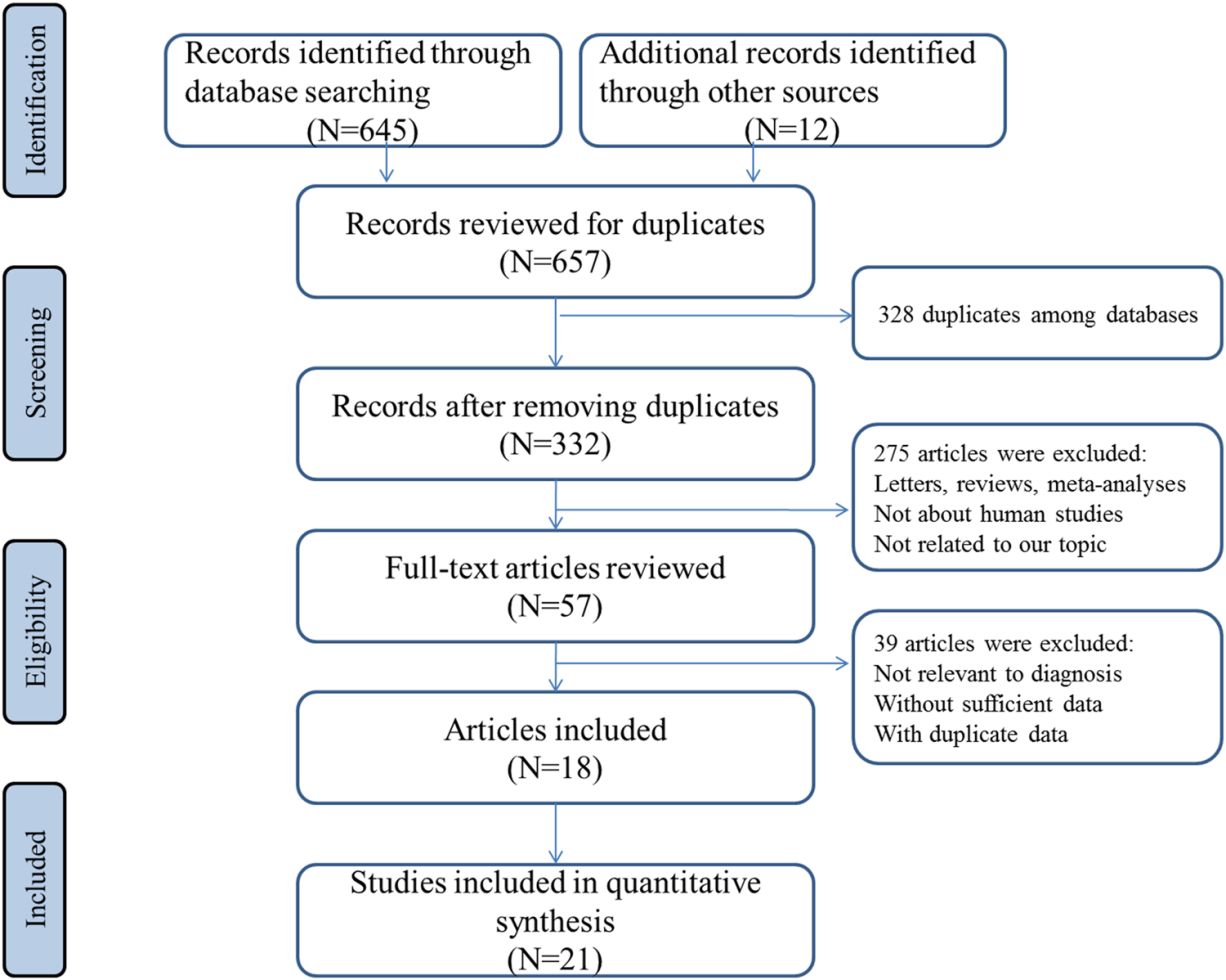
The flowchart based on the inclusion and exclusion criteria

In total, 657 potentially eligible studies were obtained from online database searching. After manually scanning the titles, abstracts and key data, 600 records were excluded because of meta-analysis, letters, reviews, duplicate studies, or their irrelevance to the present analysis. After screening full texts of the remaining 57 articles, 39 publications were further excluded because they failed to satisfy our inclusion criteria. Finally, 18 publications containing 21 studies were considered in the meta-analysis [22–39]. Our flow diagram of each stage for inclusion and exclusion was presented in Fig. 1.

The main characteristics of each enrolled publication were summarized in Table 1. The publication years of the included studies were from 2012 to 2020. All the studies performed reverse transcription quantitative PCR (RT-qPCR) for miR-155 detection, and the specimen type included serum, plasma, blood, urine, and tissue. The quality of the included articles was evaluated by querying the QUADAS-2 scores. As a result, the overall methodological qualities of the study designs of selected studies were generally good.

**Table 1:** The main features of the included studies for miR-155 in the diagnosis of breast cancer

Author	Year	Country	Ethnicity	Case			Control		Source	Methods	AUC	Sensitivity	Specificity	Score
				No	Age	Stage	No	Age						
Sun et al	2012	China	Asian	103	53	I–IV	55	51	Serum	qRT-PCR	0.801	0.65	0.82	4
Zhao et al	2012	China	Asian	20	54	I–IV	10	51	Serum	qRT-PCR	NA	0.99	0.90	4
Eichelser et al	2013	Germany	Non-Asians	152	65	I–IV	40	65	Serum	qRT-PCR	NA	0.64	0.80	5
Mar-Aguilar et al	2013	Mexico	Non-Asians	61	53	I–III	10	NA	Serum	qRT-PCR	0.994	0.95	1.00	5
Erbes et al	2015	Germany	Non-Asians	24	54	I–III	24	52	Urine	qRT-PCR	0.814	0.79	0.83	5
Shaker et al	2015	Egypt	Non-Asians	100	NA	I–IV	30	NA	Serum	qRT-PCR	0.993	0.94	1.00	4
Gao et al	2017	USA	Non-Asians	75	51	I–III	50	52	Plasma	qRT-PCR	0.77	0.64	0.78	4
Gao et al	2017	USA	Non-Asians	39	51	IV	21	47	Plasma	qRT-PCR	0.75	0.74	0.76	4
Han et al	2017	China	Asians	99	49	I–III	21	45	Serum	qRT-PCR	0.749	1.00	0.51	5
Fan et al	2018	China	Asians	49	43	I–IV	19	NA	Serum	qRT-PCR	0.793	1.00	0.60	5
Huang et al	2018	China	Asians	30	51	I–IV	30	50	Serum	qRT-PCR	0.817	0.83	0.80	5
Huang et al	2018	China	Asians	128	51	I–IV	70	50	Serum	qRT-PCR	0.638	0.41	0.87	5
Zaleski et al	2018	Germany	Non-Asians	33	59	I–IV	19	44	Serum	qRT-PCR	0.525	0.16	0.24	5
Arabkari et al	2019	Ireland	Non-Asians	38	NA	NA	20	NA	Blood	qRT-PCR	0.795	0.95	0.70	4
Soleimanpour et al	2019	Iran	Asians	30	50	I–III	30	50	Tissue	qRT-PCR	0.83	0.86	0.74	5
Soleimanpour et al	2019	Iran	Asians	30	50	I–III	25	50	Plasma	qRT-PCR	0.92	0.87	0.88	5
Song et al	2019	China	Asians	64	49	I–IV	58	49	Plasma	qRT-PCR	0.88	0.96	0.66	4
Swellam et al	2019	Egypt	Non-Asians	96	50	I–IV	39	53	Serum	qRT-PCR	0.995	0.96	0.96	5
Swellam et al	2019	Egypt	Non-Asians	80	52	I–III	30	52	Serum	qRT-PCR	0.993	0.95	0.97	5
Bitaraf et al	2020	Iran	Asians	50	50	I–IV	50	50	Tissue	qRT-PCR	0.74	0.84	0.54	4
Raeisi et al	2020	Iran	Asians	15	47	I–III	15	47	Tissue	qRT-PCR	0.941	0.84	0.91	4

*AUC* area under curve, N*A* not available

### Diagnostic performance of miR-155 in BC

**Fig. 2:**
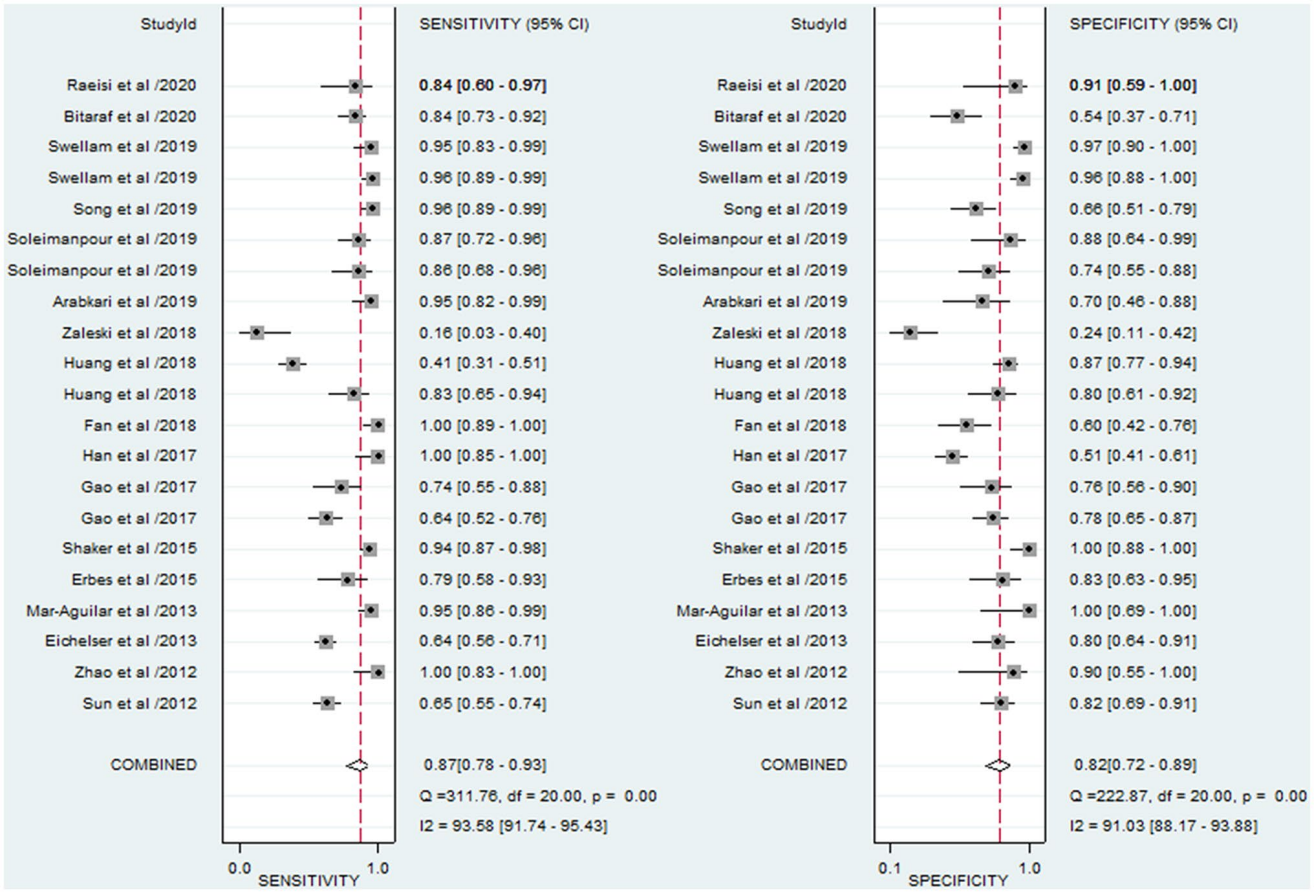
Forest plots of sensitivity and specificity for miR-155 test in breast cancer

**Fig. 3:**
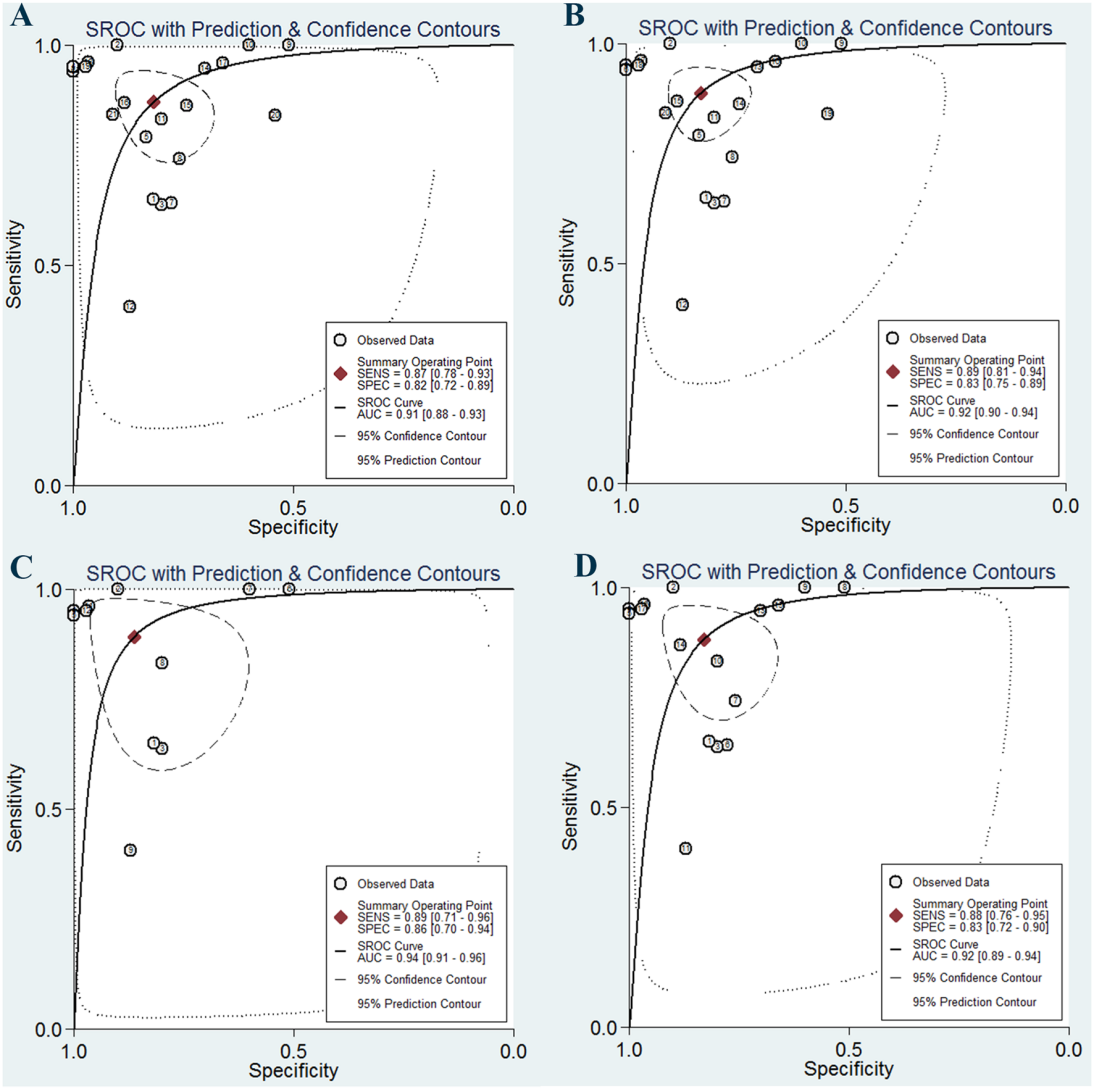
The SROC curves of the pooled individual analyses. **a** Combined analyses; **b** outliers excluded; **c** serum samples; **d** circulating samples

The forest plots of sensitivity and specificity of miR-155 in the diagnosis of BC are presented at Fig. 2. The overall analysis indicated the pooled sensitivity and specificity were 0.87 (95% CI 0.78–0.93) and 0.82 (95% CI 0.72–0.89), respectively. We also noted that the pooled PLR was 4.8 (95% CI 3.0–7.7), NLR was 0.16 (95% CI 0.09–0.29), and DOR was 30 (95% CI 11–80). The SROC curve for the enrolled diagnostic tests is shown at Fig. 3. The corresponding AUC was 0.91 (95% CI 0.88–0.93), revealing that miR-155 has relatively high diagnostic accuracy for BC.

### Test of heterogeneity and subgroup analysis

The Spearman correlation coefficient was 0.35 with a *P* value of 0.12, indicating no obvious heterogeneity from threshold effect in the present data pooling.

The *I*^2^ values of sensitivity and specificity were 93.58% (95% CI 91.74–95.43%; *P* < 0.01) and 91.03% (95% CI 88.17–93.88%; *P* < 0.01), respectively, indicating significant heterogeneity from non-threshold effect in our study. To investigate the between-study heterogeneity, subgroup analyses based on ethnicity (Asian or non-Asian), sample size (*N* > 100 or *N* < 100) and sample source (serum, plasma and tissue) were conducted (Table 2). A comparison of sample size in large sample size (*N *> 100) and small sample size (*N* < 100) indicated that the specificity (0.85 versus 0.77), DOR (36 versus 24), PLR (5.7 versus 3.8), and AUC (0.92 versus 0.89) were higher in studies with large sample size than studies with small sample size, suggesting that large cohort studies are warranted and worthwhile. For studies on Asian populations, the pooled sensitivity was 0.90, specificity was 0.73, PLR was 3.3, NLR was 0.14, DOR was 23, and AUC was 0.84. For studies on non-Asian populations, the pooled sensitivity was 0.83, specificity was 0.87, PLR was 6.7, NLR was 0.19, DOR was 35, and AUC was 0.92. The sensitivity of studies using plasma was 0.84 and the specificity was 0.75, with a pooled DOR of 15 and AUC of 0.80. Nevertheless, for serum-based studies, the sensitivity of studies was 0.89 and the specificity was 0.86, with a pooled DOR of 52 and AUC of 0.94, indicating that the use of serum miR-155 as relatively reliable matrix in diagnosing BC. Since most sample sources concentrated on circulating samples, we performed subgroup analysis based on this point. The results indicated that the pooled sensitivity, specificity, PLR, NLR, DOR, and AUC (Fig. 3) for circulating miR-155 in the detection of BC were 0.88 (95% CI 0.76–0.95), 0.83 (95% CI 0.72–0.90), 5.2 (95% CI 2.9–9.3), 0.14 (95% CI 0.06–0.31), 37 (95% CI 11–123) and 0.92 (95% CI 0.89–0.94), respectively.

**Table 2:** Summary table of the diagnostic accuracy of miR-155 for breast cancer

Analyses	Studies	Sensitivity (95% CI)	Specificity (95% CI)	DOR (95% CI)	PLR (95% CI)	NLR (95% CI)	AUC (95% CI)
Sample types							
Serum	12	0.89 (0.71–0.96)	0.86 (0.70–0.94)	52 (9–306)	6.5 (2.7–16.0)	0.13 (0.04–0.38)	0.94 (0.91–0.96)
Plasma	4	0.84 (0.66–0.93)	0.75 (0.66–0.82)	15 (6–38)	3.3 (2.5–4.4)	0.22 (0.10–0.48)	0.80 (0.77–0.84)
Circulating	17	0.88 (0.76–0.95)	0.83 (0.72–0.90)	37 (11–123)	5.2 (2.9–9.3)	0.14 (0.06–0.31)	0.92 (0.89–0.94)
Ethnicity							
Asians	11	0.90 (0.77–0.96)	0.73 (0.64–0.80)	23 (10–51)	3.3 (2.6–4.3)	0.14 (0.06–0.32)	0.84 (0.81–0.87)
Non-Asians	10	0.83 (0.65–0.93)	0.87 (0.73–0.95)	35 (6–128)	6.7 (2.5–17.4)	0.19 (0.08–0.47)	0.92 (0.90–0.94)
Sample size							
Large sample size (> 100)	10	0.86 (0.72–0.94)	0.85 (0.71–0.93)	36 (10–132)	5.7 (2.7–11.9)	0.16 (0.07–0.36)	0.92 (0.90–0.94)
Small sample size (< 100)	11	0.87 (0.74–0.95)	0.77 (0.64–0.87)	24 (6–95)	3.8 (2.1–6.9)	0.16 (0.07–0.39)	0.89 (0.85–0.91)
Overall	21	0.87 (0.78–0.93)	0.82 (0.72–0.89)	30 (11–80)	4.8 (3.0–7.7)	0.16 (0.09–0.29)	0.91 (0.88–0.93)
Outliers excluded	20	0.89 (0.81–0.94)	0.83 (0.75–0.89)	39 (18–84)	5.2 (3.5–7.9)	0.13 (0.08–0.23)	0.92 (0.90–0.94)

*DOR* diagnostic odds ratio, *PLR* positive likelihood ratio, *NLR* negative likelihood ratio, *AUC* area under curve

Subsequently, we attempted to explain the heterogeneity by exploring some study characteristics including sample size, sample source, and ethnicity, through meta-regression analyses. However, no significant results were identified.

### Sensitivity analysis and publication bias

**Fig. 4:**
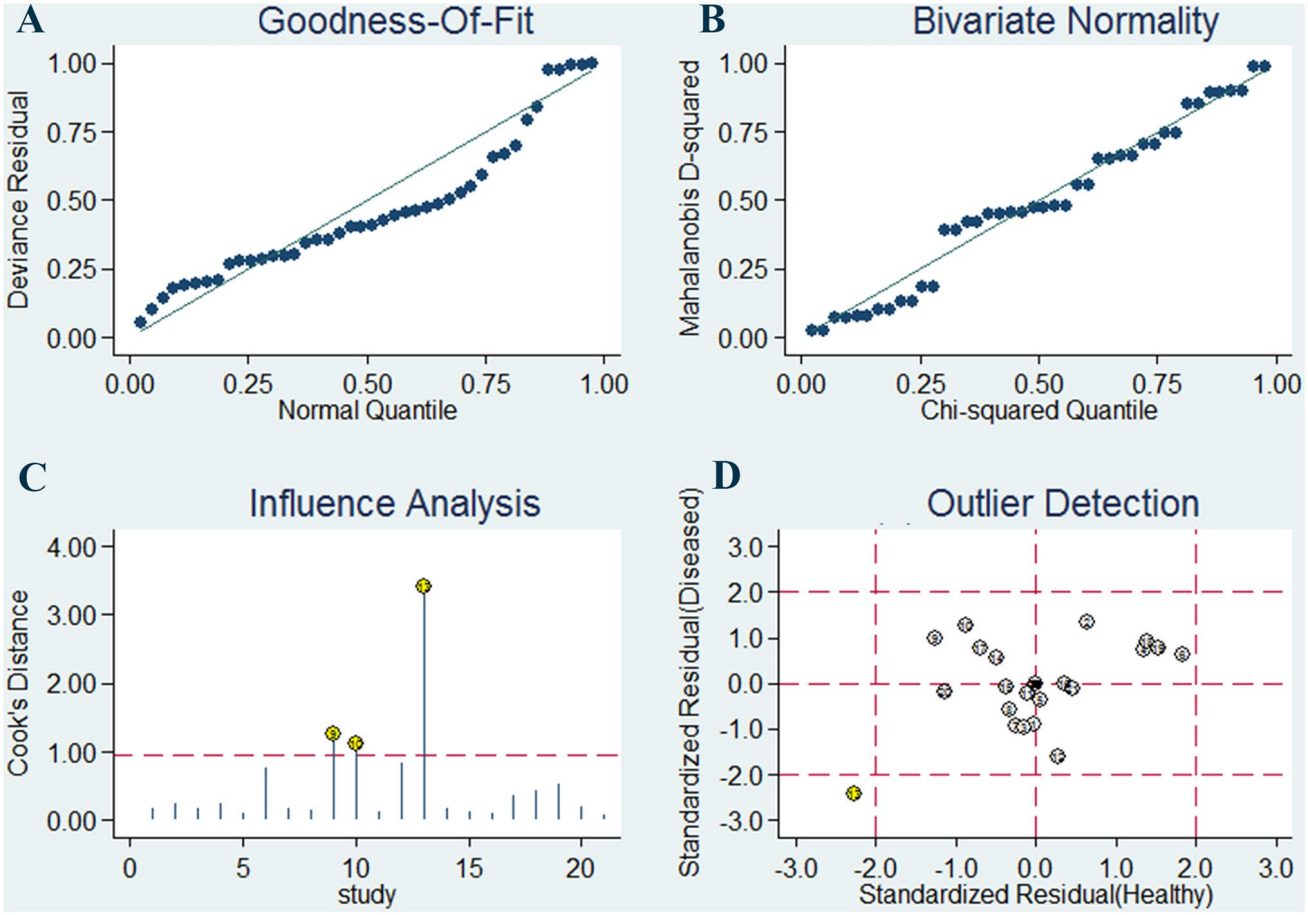
Sensitivity analysis results. **a** Goodness-of-fit; **b** bivariate normality; **c** influence analysis; **d** outlier detection

Afterwards, influence analysis was employed to evaluate the influence of single studies on overall estimates (Fig. 4). The random-effect bivariate model was demonstrated to be robust for the calculation of the pooled parameters by conducting the goodness of fit and bivariate normality analyses. From the illustrated results, a study from Zaleski et al. [31] was identified as an outlier. After excluding the outlier, the overall pooled sensitivity for miR-155 increased from 0.87 to 0.89, specificity increased from 0.82 to 0.83, DOR increased from 30 to 39, PLR increased from 4.8 to 5.2, NLR decreased from 0.16 to 0.13, and AUC increased from 0.91 to 0.92. Moreover, the *I*^2^ values for sensitivity and specificity were not significantly influenced by the outlier study. In general, the overall results did not show significant changes, indicating that the overall pooled estimates could not be affected by a single study.

**Fig. 5:**
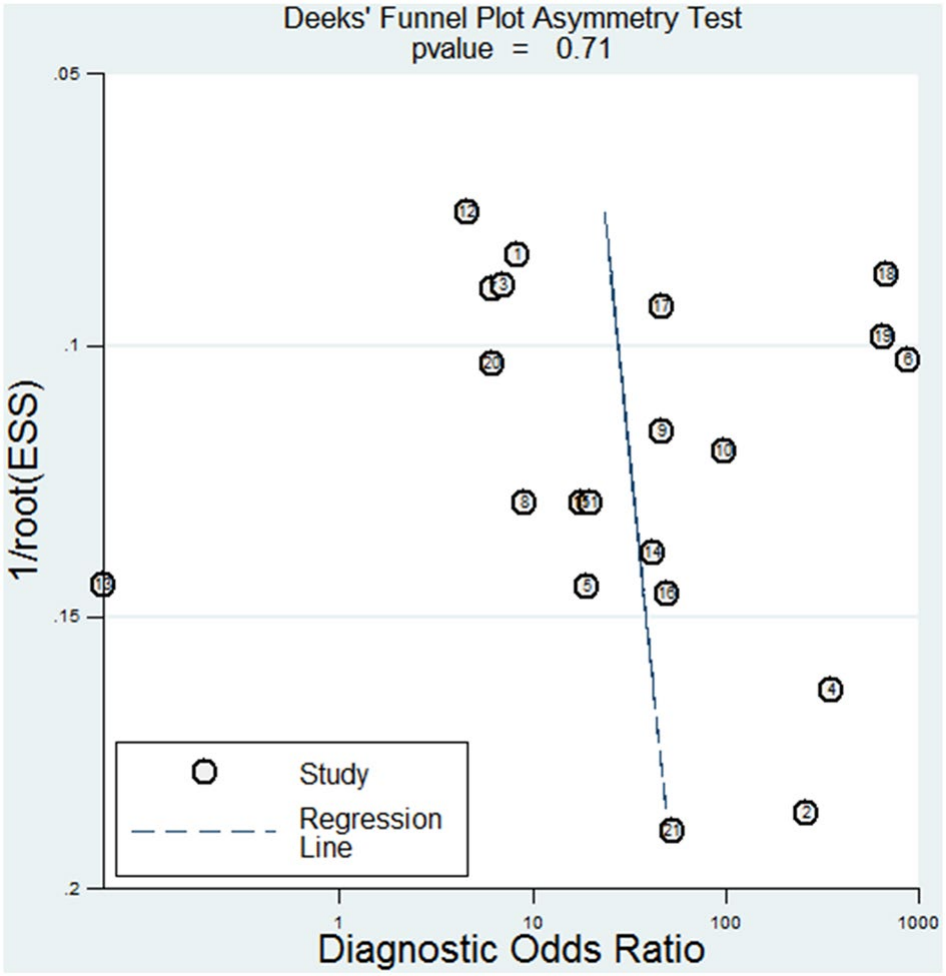
The publication bias of all included studies

We then used the Deeks’ funnel plot to check for publication bias. The slope coefficient presented a *P* value of 0.71 (Fig. 5), revealing a low likelihood of publication bias.

### Functional and pathway enrichment analyses

**Fig. 6:**
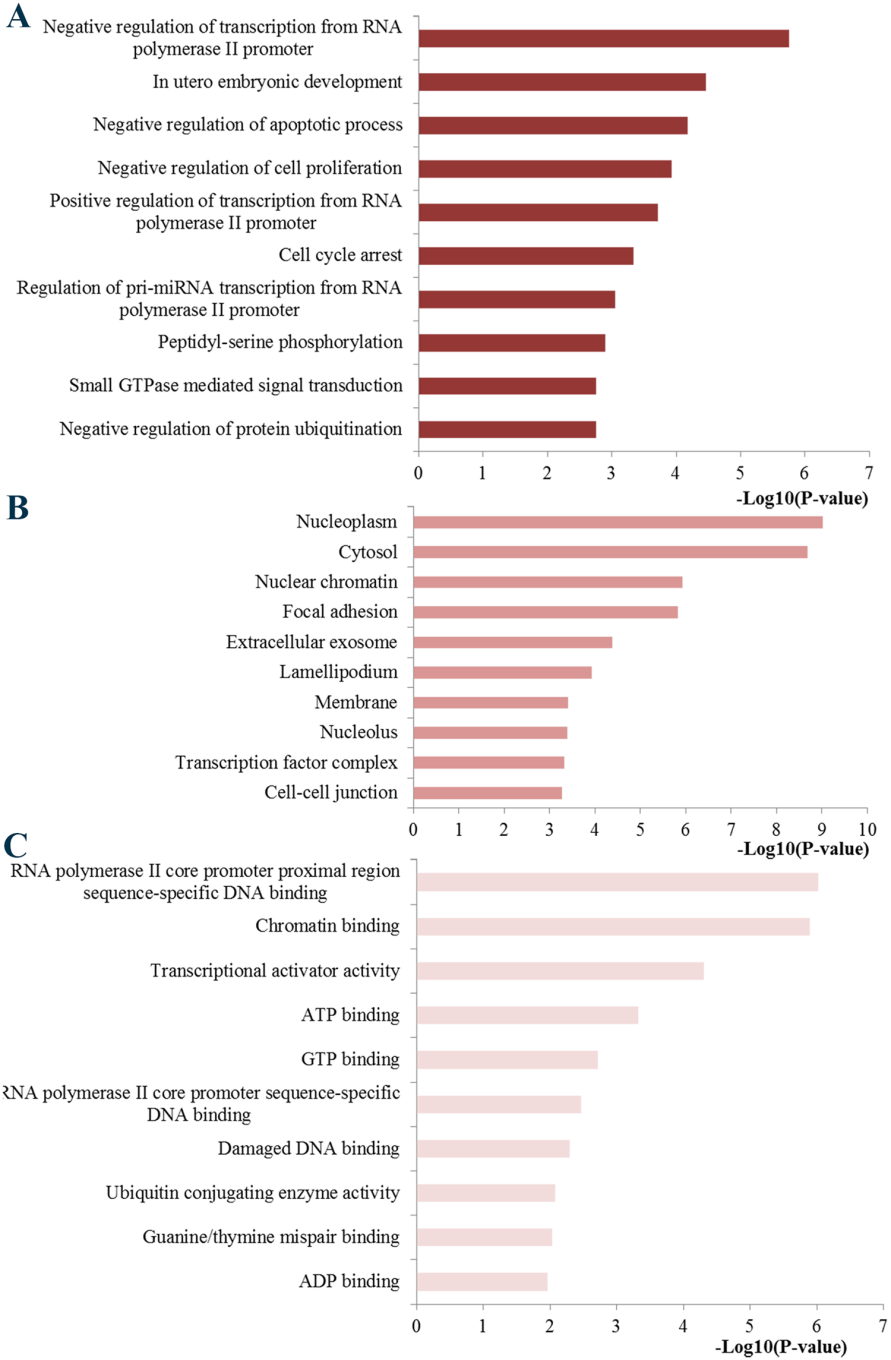
Top 10 of the most significantly enriched GO terms. **a** Biological process; **b** cellular component; **c** molecular function. GO, Gene Ontology

GO functional annotation and KEGG signaling pathway enrichment analysis were performed with the genes regulated by miR-155 to obtain an in-depth and comprehensive understanding of the biological activities of miR-155. The target genes of miR-155 were retrieved from miRTarBase and then conducted with functional GO enrichment analysis. GO analysis results indicated that BP terms of the PPI network were significantly enriched in cell cycle arrest, regulation of pri-miRNA transcription, peptidyl-serine phosphorylation, small GTPase-mediated signal transduction and negative regulation of protein ubiquitination, CC terms in nucleoplasm, cytosol, nuclear chromatin, focal adhesion and extracellular exosome, and MF terms in RNA polymerase II core promoter proximal region sequence-specific DNA binding, chromatin binding, ATP binding and GTP binding (Fig. 6).

**Fig. 7:**
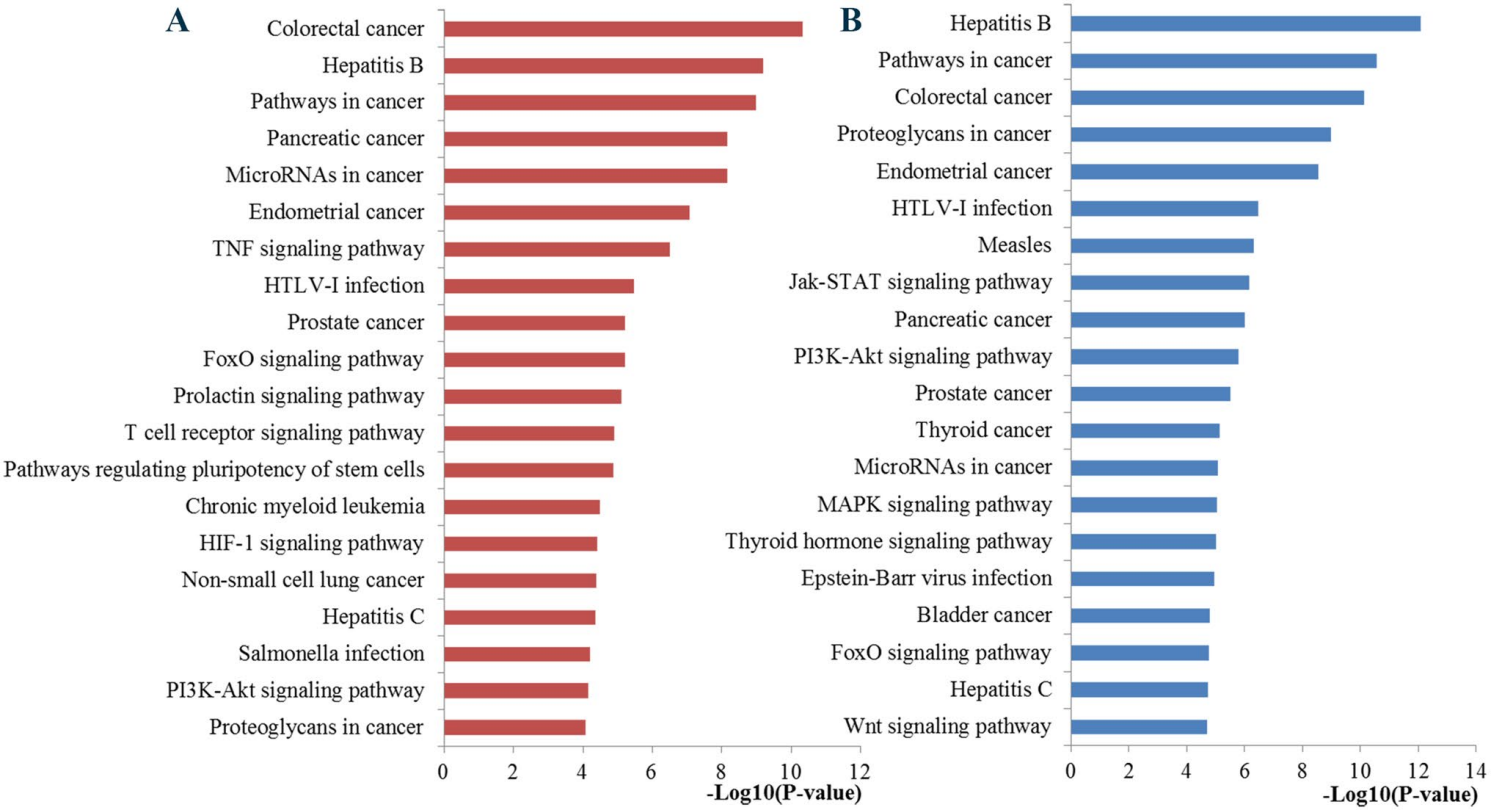
Significantly enriched KEGG pathway. **a** Top 20 pathways enriched by all the targets of miR-155; **b** top 20 pathways enriched by the ten hub genes

**Fig. 8:**
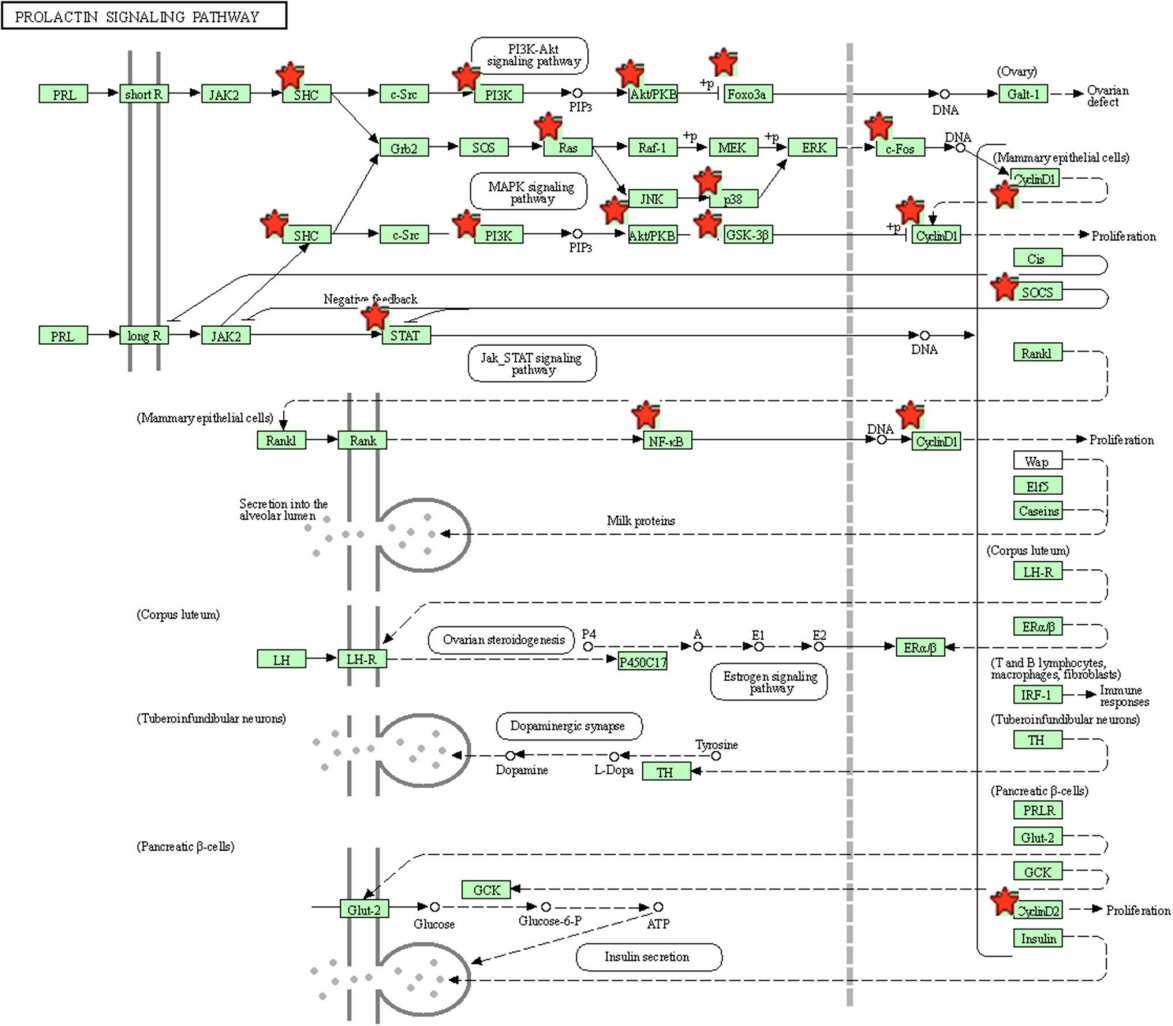
The prolactin pathway. The positions in which the target genes of miR-155 cause action are shown with red stars

KEGG pathway enrichment analysis indicated that the genes regulated by miR-155 were significantly enriched in pathways in cancer, microRNAs in cancer, TNF signaling pathway, FoxO signaling pathway, prolactin signaling pathway, T cell receptor signaling pathway, signaling pathways regulating pluripotency of stem cells, HIF-1 signaling pathway, and PI3K-Akt signaling pathway (Fig. 7). According to previous studies and KEGG results, it could be speculated that the prolactin signaling pathway may be one of the most important pathways. The chart arising from the KEGG pathway analysis is presented at Fig. 8.

### PPI network integration and selection of hub genes

**Fig. 9:**
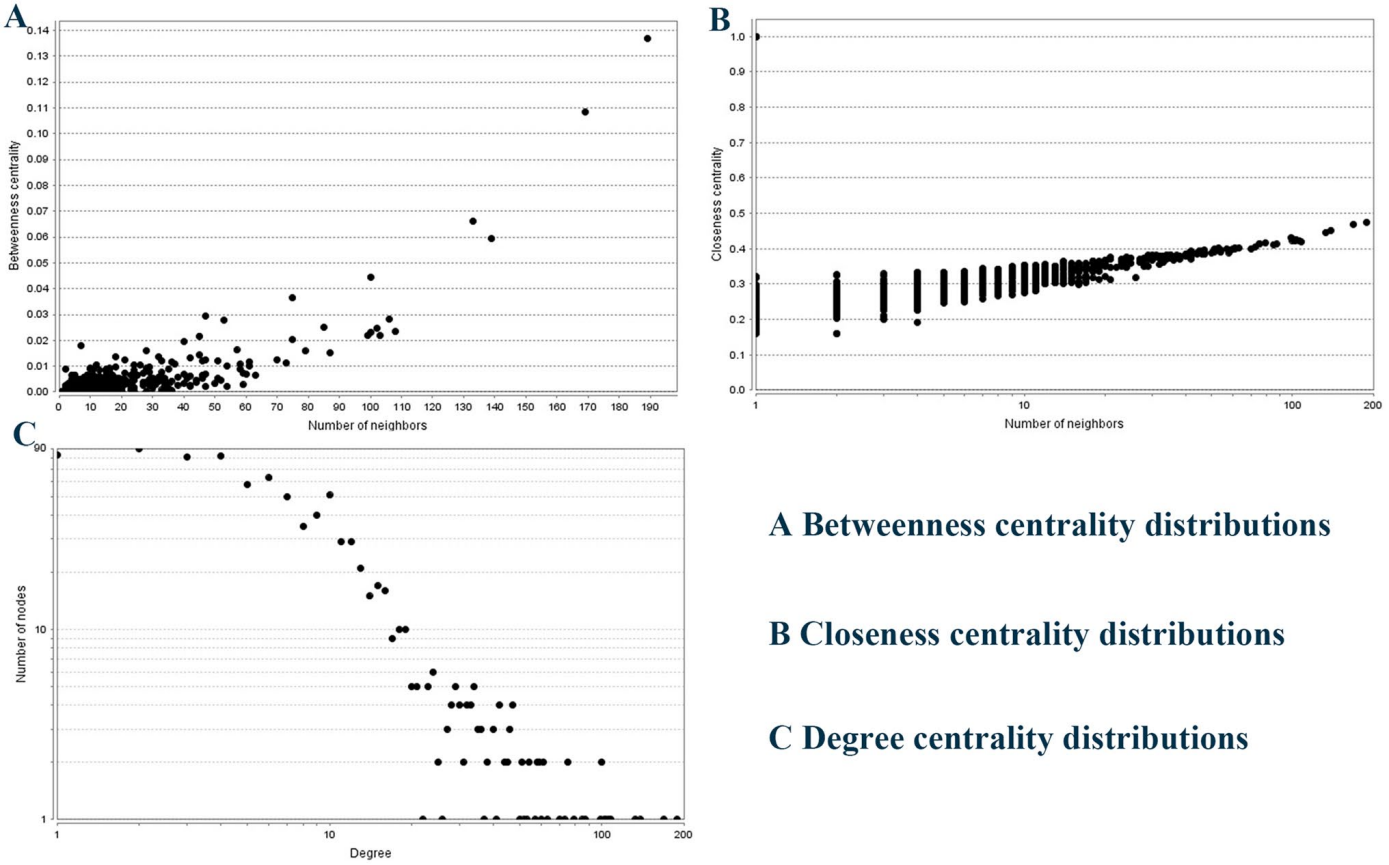
PPI network analysis results. PPI, protein–protein interaction

We applied the STRING online database to analyze the 965 identified target genes of miR-155 and to set up a PPI network, which consisted of 907 nodes interacting with the 11.742 average numbers of neighbors. The results were downloaded and visualized by Cytoscape software for further analysis. The hub genes were selected by CytoNCA plug-in, and the top significant hub genes were obtained by betweenness centrality, closeness centrality and degree centrality methods, respectively (Fig. 9). At last, 10 hub genes were identified, including TP53, AKT1, EGFR, MYC, CTNNB1, IL6, JUN, STAT3, CASP3, and CCND1. To clarify the signal pathways associated with the hub genes during the initiation and progression of BC, we performed KEGG pathway enrichment analysis using DAVID software. The results in Fig. 7 disclosed the most vital KEGG pathways of the hub genes regulated by miR-155 associated with BC including pathways in cancer, proteoglycans in cancer, Jak-STAT signaling pathway, PI3K-Akt signaling pathway, microRNAs in cancer, MAPK signaling pathway, and FoxO signaling pathway.

### Module analysis

**Fig. 10:**
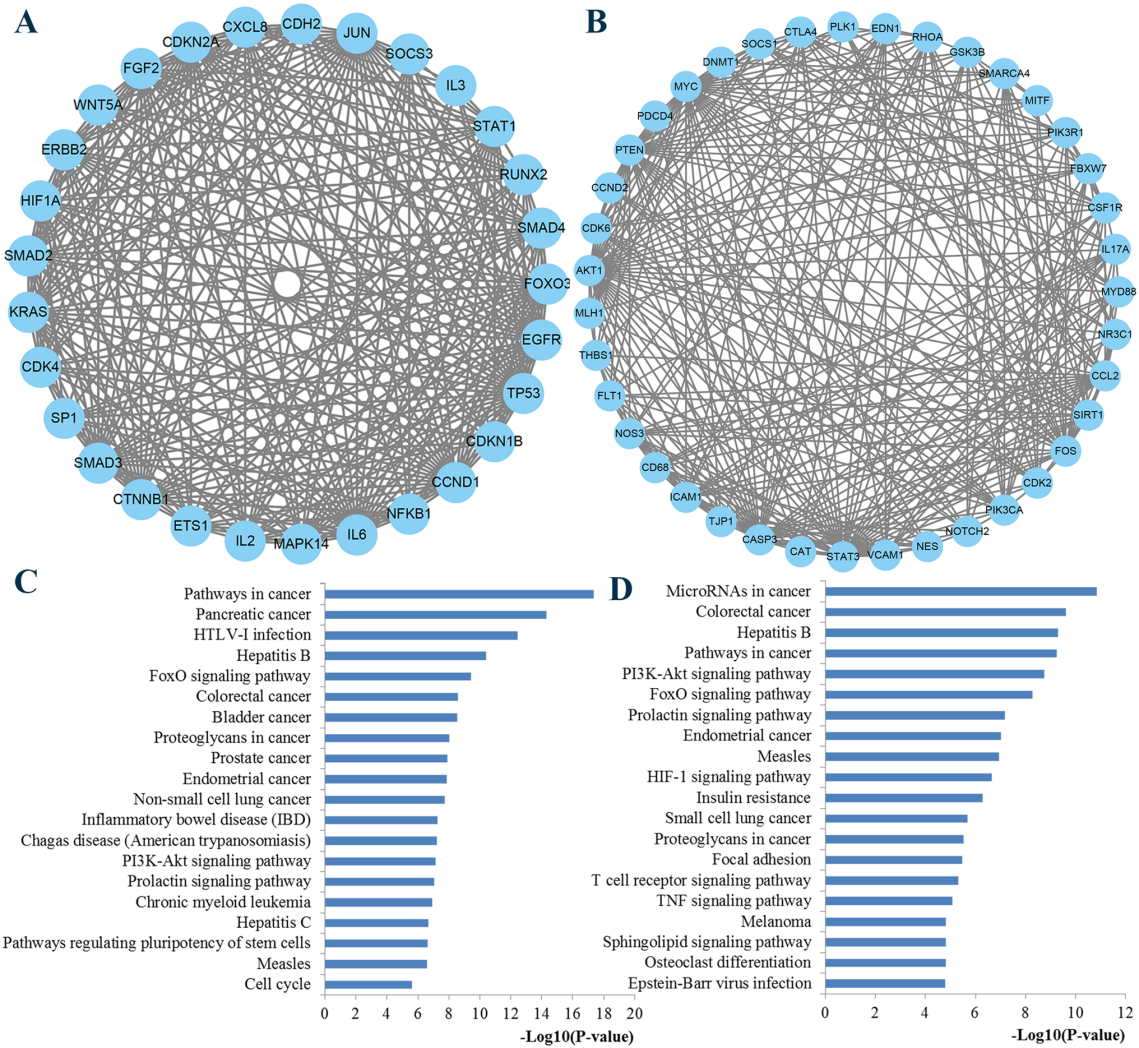
Module analysis from the protein–protein interaction network. **a**,** b** Top two modules extracted from the PPI network; **c, d** pathway enrichment analysis of the genes in the top two modules

Based on the MCODE plug-in, the top two significant modules of the above PPI network were identified and reconstructed with Cytoscape (Fig. 10). Then, KEGG pathway analysis was conducted to predicate the potential module function and cellular pathways. A list of pathways that were notably enriched by the module nodes were pathways in cancer, FoxO signaling pathway, proteoglycans in cancer, PI3K-Akt signaling pathway, prolactin signaling pathway, cell cycle, microRNAs in cancer, HIF-1 signaling pathway, focal adhesion, T cell receptor signaling pathway, TNF signaling pathway, and sphingolipid signaling pathway (Fig. 10).

## Discussion

Sensitive and specific tumor biomarkers are vital for early cancer detection and diagnosis and for adopting novel therapeutic trials and prevention strategies in clinic. Increasing evidence has demonstrated that microRNAs can take part in various critical processes containing tumor cell mutation, proliferation, invasion, progression and metastasis. Abnormal microRNA expression has been identified in extensive researches, and microRNAs could function as tumor suppressor or promoter in a variety of cancers. Among all studied microRNAs, miR-155 is no doubt one of the most attractive members, which has been explored to be a potential valuable biomarker for cancer detection and survival prediction. Nevertheless, the sample sizes in most studies are relatively small. Moreover, it is still inconclusive regarding the diagnostic roles of miR-155 expression in BC. In the present study, we systematically reviewed clinical studies on the application of miR-155 for diagnosis of BC in recent years and performed this study to clarify the clinical significance of miR-155 expression in BC.

In the comprehensive analysis of diagnostic accuracy of miR-155 and BC, the pooled sensitivity and specificity of miR-155 in the detection of BC was 0.87 and 0.82, respectively. The AUC, which is recommended for evaluating the accuracy of a diagnostic test, was calculated to be 0.91, suggesting that miR-155 has relatively high clinical significance for diagnosis of BC. The value of a DOR is also an indicator of test performance; it ranges from 0 to infinity, with higher values suggesting better test identification. The pooled DOR was 30 in our study, revealing that miR-155 assays had excellent test performance in the diagnosis of BC. The overall PLR value was 4.8, revealing that the probability of having BC in a person with a positive test result was approximately fivefold higher compared to non-cancer patients. The pooled NLR was 0.16, meaning that the probability of a patient having BC is 16% if the miR-155 assay exhibits a negative result. Taken together, these results suggest a higher level of accuracy and better diagnostic characteristics for miR-155 in the diagnosis of BC than traditional detection methods.

To explore the potential sources of the heterogeneity, we carried out subgroup analysis and meta-regression analysis. Our subgroup analysis based on the sample size indicated that miR-155 with large sample size had a higher accuracy than miR-155 with small sample size, suggesting a large samples of clinical study is needed. Meanwhile, the subgroup analyses based on specimen types indicated that serum had relatively higher diagnostic value compared to plasma, indicating serum may be applied as more suitable sources of clinical specimens than others for BC detection. However, this should be interpreted with caution as levels could differ significantly if the source was serum versus plasma, because of coagulation processes. More studies should be conducted to further explore the most suitable sources of specimens. Due to the genetic heterogeneity, a difference in diagnostic accuracy has been displayed among different ethnicities. The meta-regression result revealed that these factors may not be the potential sources contributing to heterogeneity in this study. In addition, the sensitivity analysis indicated that the overall pooled estimates could not be affected by a single study.

Currently, some efforts have been devoted to exploring the functional roles of miR-155 in the pathogenic mechanism and malignant progression of cancer. However, the underlying molecular mechanisms involved in cancer progression are still largely unclear. Thus, we used some bioinformatics analysis methods to explore the functional activities of miR-155 targets involved in the occurrence and development of BC. GO analysis indicated that miR-155 was associated with some vital biological processes, basic cell components and the binding function of some key molecules. Moreover, KEGG functional enrichment analysis identified a series of important pathways, which are closely associated with the initiation and progression of BC. For example, pathways in cancer and microRNAs in cancer directly reflected the associations between miR-155 and some vital pathways involved in BC. TNF signaling has been one of the most significant cellular pathways with important functions in homeostasis and disease pathogenesis [40]. Studies have convinced the roles of FoxO signaling pathway in regulating genes essential for cell proliferation, cell death, senescence, angiogenesis, cell migration and metastasis [41]. The prolactin pathway, known as a hormone involved in normal breast development and lactation, has been long demonstrated to be involved in the progression of human breast cancer [42]. It has been confirmed that T cell receptor signaling pathway has important roles in the host adaptive immune system and T cell-based adoptive immunotherapy has been shown to be a promising treatment for various types of cancers. The abnormal expression of T cell receptor may lead to carcinogenesis [43]. HIF-1 has been recognized as an essential component in changing the transcriptional response of tumors under hypoxia and plays important roles in crucial aspects of cancer biology, including angiogenesis, cell survival, glucose metabolism and invasion [44]. PI3K-Akt signaling pathway was demonstrated to be hyperactivated in most of the breast carcinomas and could regulate various biological processes such as cell growth, differentiation, migration, and survival, as well as angiogenesis and metabolism [45]. Moreover, previous evidence has reported that microRNAs have critical roles in cellular activities implicated in BC cell growth, migration and metastasis by targeting the PI3K/AKT oncogenic signaling pathway [46]. These functional enrichment results may help us understand the mechanisms of miR-155 during the initiation and progression of BC.

PPI analysis has been recognized as an entry point for better interpretation of associations between different proteins on a genome-wide scale, and might be helpful to provide novel insights into protein function explanation. We constructed the PPI network with the target genes of miR-155 and identified the top ten hub genes. These hub genes were predominantly associated with some important pathways, most of which have been confirmed to be related to BC. In addition, there is growing evidence that proteoglycans can adjust extensive normal and pathological activities, such as morphogenesis, tissue repair, inflammation, vascularization and cancer metastasis [47]. Rapidly emerging ground-breaking discoveries have revealed that Jak-STAT signaling pathway regulates almost all immune regulatory processes, containing those that are involved in tumor cell recognition and tumor-driven immune escape [48]. Alterations in this pathway may contribute to the development of BC and metastatic spread and targeting of JAK-STAT signaling in BC may provide potential therapeutic strategies to overcome drug resistance [49]. The MAPK signaling pathway plays an important part in organizing great constitution network that mediates a variety of physiological processes, such as cell growth, differentiation, as well as apoptotic cell death and dysregulation of the MAPK signaling cascades has been confirmed to be associated with the pathogenesis of various human cancer types including BC [50, 51]. The hub genes may provide potential predictor and therapeutic targets for BC patients.

The module analysis of the constructed PPI network was then carried out and the top two significant modules were selected for conducting KEGG pathway enrichment analysis of the genes included. The enriched results indicated that the nodes involved in the modules were highly related to some vital pathways. In addition to the most pathways which were already discussed above, cell cycle has been identified as a hallmark of cancer involved in cellular proliferation and cancer development. The dysregulation of cell cycle control may lead to cancer progression [52]. Moreover, focal adhesion kinase (FAK) is a cytoplasmic non-receptor tyrosine kinase involved in almost every aspect of cancer, from invasion to metastasis to epithelial–mesenchymal transition (EMT) and maintenance of cancer stem cells [53]. Sphingolipids have been the largest family of bioactive lipids that participate in a wide variety of biological mechanisms, including cell death and proliferation and are linked with numerous aspects of tumorigenesis [54]. The PPI analysis further uncovered the potential mechanisms of miR-155 involved in BC occurrence and development. However, further studies are still urgently demanded to validate the hub genes and pathways, and deeper mechanisms would be uncovered.

We should point out that there are some limitations in our study. First, the cutoff values of miR-155 in the enrolled diagnostic tests were not uniform, which might contribute to the observed heterogeneity. More studies should be conducted to reach an appropriate cutoff value to be accurate enough. Second, the sample sizes for plasma, whole blood, tissue and urine are relatively small. It is difficult to make a definitive conclusion about the accuracy of miR-155 in diagnosing BC from these sample sources. Thus, further evaluations of the utility of miR-155 from different sample sources as biomarkers for BC detection should be performed. Third, the lack of studies for different ethnicities evaluating the miR-155 diagnostic performance complicated the exploration of its clinical application. More studies should be conducted to evaluate the diagnostic accuracy of miR-155 for different ethnicities. In addition, due to the fact that all studies that are used for the data pooling have patients of various TNM stage (from stage I to stage IV), there is considerable heterogeneity in detection of BC. Without patient-level data, it would be very difficult to tell whether miR-155 is actually useful as a marker in early detection of BC. Besides, BRCA1-deficient tumors have been shown to have increased levels of miR-155, which could be relevant for identifying possible respondents to PARP-1 inhibitors [55, 56]. Most of the mechanistic studies on miR-155 and BC have been done on these patients, which was ignored in this study. Last, due to lack of experiment verification, the function and mechanisms of miR-155 need to be further studied in vivo and in vitro.

Despite all this, we revealed that miR-155 had great potential in early diagnosis for BC and further studies to explore its clinical application are warranted and worthwhile. Some interesting findings will stimulate further research with rigid criteria and large study populations to resolve any remaining controversy of the diagnostic value of miR-155 in BC patients. The bioinformatics analysis results definitely point out the molecular basis to better understand the pathogenesis of miR-155 involved in BC and provide valuable novel markers or targets for the diagnosis and treatment of BC.

## Conclusion

In conclusion, the present study according to published articles demonstrated that miR-155 has strong potential to serve as a novel noninvasive biomarker for detection of BC. In addition, integrated bioinformatics analysis identified some key hub genes and pathways, which may help understand why miR-155 could possess excellent biomarker characteristics and provide some new clues to explore the exact etiology and mechanism underlying the initiation and progression of BC. To strengthen our findings, the diagnostic value of miR-155 in BC should be further confirmed by large-scale and standard investigations. Moreover, further studies are urgently demanded to validate the hub genes and pathways, and further mechanisms would be uncovered.

## Data Availability

The data supporting the conclusions of this article are within the article.

## Abbreviations

BC: Breast cancer
AUC: Area under the curve
TP: True positive
FP: False positive
FN: False negative
TN: True negative
PLR: Positive likelihood ratios
NLR: Negative likelihood ratios
DOR: Diagnostic odds ratio
CI: Confidence interval
SROC: Summary receiver operator characteristic
QUADAS-2: Quality Assessment of Diagnostic Accuracy Studies 2
GO: Gene ontology
KEGG: Kyoto Encyclopedia of Genes and Genomes
PPI: Protein–protein interaction
DAVID: Database for Annotation, Visualization, and Integrated Discovery
